# Expanding the Phenotypic Spectrum of Anauxetic Dysplasia Type 3: Reporting an Iranian Family With Unique Systemic Features and *NEPRO* Gene Variant

**DOI:** 10.1002/ccr3.72355

**Published:** 2026-04-12

**Authors:** Mahnaz Mohammadi Kian, Sara Sheikholeslami, Maryam Kiani Feizabadi, Amir Mohammad Roshanaeizadeh, Atousa Haghi, Ahmad Reza Salehi Chaleshtori, Hamid Reza Moazzeni

**Affiliations:** ^1^ Momgene Personalized Medicine Center Tehran Iran; ^2^ Department of Medical Genetics, School of Medicine Tehran University of Medical Sciences Tehran Iran; ^3^ Research Institute for Oncology, Hematology and Cell Therapy Tehran University of Medical Sciences Tehran Iran

**Keywords:** anauxetic dysplasia type 3, cartilage hair hypoplasia‐anauxetic dysplasia, *NEPRO* gene, whole exome sequencing

## Abstract

The cartilage hair hypoplasia‐anauxetic dysplasia (CHH‐AD) spectrum represents a group of rare autosomal recessive skeletal dysplasias with significant phenotypic heterogeneity. These disorders are classified based on pathogenic variants in the *RMRP*, *POP1*, and *NEPRO* genes. Among these, anauxetic dysplasia type 3 (ANXD3), associated with *NEPRO* variants, manifests as severe skeletal dysplasia characterized by short stature, brachydactyly, skin laxity, and joint hypermobility, with distinct radiographic findings such as ovoid vertebrae, hypoplastic ilia, narrow acetabular angles, and irregular metaphyses. Unlike other CHH‐AD subtypes, ANXD3 lacks immunological or gastrointestinal involvement. This study reports three new ANXD3 cases from a consanguineous Iranian family, carrying the homozygous pathogenic variant Chr3:113014014G>A; exon3; c.280C>T; p.Arg94Cys in the *NEPRO* gene. The clinical phenotypes expand the known spectrum of ANXD3, including unique features such as microcephaly, clubfoot, cataracts, urolithiasis, and hearing impairments, whi12‐18ch suggest systemic involvement beyond skeletal abnormalities. Diagnostic whole‐exome sequencing, supported by Sanger validation, confirmed the autosomal recessive inheritance pattern. A comparative analysis with previously reported ANXD3 cases revealed shared characteristics, including short stature, brachydactyly, and thoracolumbar kyphoscoliosis, while highlighting variability in head size, scalp hair, and systemic features. Microcephaly was observed in our patients, same as previously reported cases, underscoring the phenotypic variability of ANXD3. This study also emphasizes the importance of genetic counseling and early interventions for associated complications, such as orthopedic, renal, and ophthalmological management.

## Key Clinical Message

ANXD3, caused by biallelic *NEPRO* variants, primarily manifests as severe skeletal dysplasia; however, our findings reveal additional systemic features—including microcephaly, cataracts, urolithiasis, and hearing loss—that expand the phenotypic spectrum. Early genetic diagnosis and multidisciplinary management are essential for improving clinical outcomes.

## 1 Introduction

Anauxetic dysplasia represents a rare group of autosomal recessive skeletal dysplasias characterized by extreme short stature and disproportionate skeletal involvement [1]. Among its subtypes, anauxetic dysplasia type 3 (ANXD3) is one of the least frequently reported and is caused by biallelic pathogenic variants in the *NEPRO* gene [2].

ANXD3 belongs to the broader spectrum of disorders historically grouped with cartilage‐hair hypoplasia‐related conditions; however, unlike classic cartilage‐hair hypoplasia, ANXD3 primarily manifests as a severe skeletal phenotype without consistent extraskeletal involvement [3, 4]. Affected individuals typically present with profound growth failure, brachydactyly, joint laxity, and characteristic radiographic abnormalities affecting the spine, pelvis, and long bones [3].

The *NEPRO* gene encodes a protein that has been implicated in RNA‐processing pathways through interactions with components of the RNase MRP complex [5]. While defects in RNA‐processing machinery are known to underlie several skeletal dysplasias, the precise mechanisms by which *NEPRO* variants lead to the clinical manifestations of ANXD3 remain incompletely understood.

To date, only a limited number of individuals with molecularly confirmed ANXD3 have been described in the literature [2, 3, 4]. Here, we report three affected individuals from a single consanguineous family harboring a homozygous pathogenic *NEPRO* variant. In addition to core skeletal features, these patients exhibit clinical findings that have been infrequently or not previously reported in ANXD3, thereby contributing to a more comprehensive delineation of the phenotypic spectrum of this ultra‐rare disorder.

## 2 Case History/Examination

**FIGURE 1:**
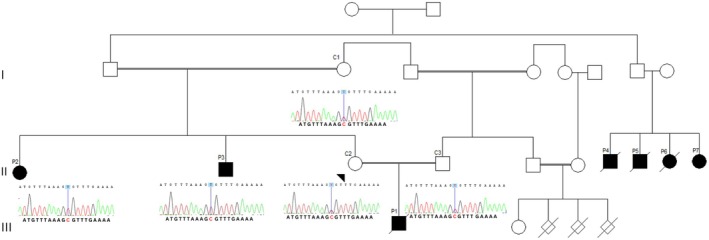
The Pedigree of this family and Sanger validation of the c.280C>T variant in the NEPRO gene (NM_015412.4). The variant is observed in the homozygous state in three affected Ps (III‐1, II‐2, and II‐3) and the heterozygous state in C (I‐1, II‐2, and II‐3).

**FIGURE 2:**
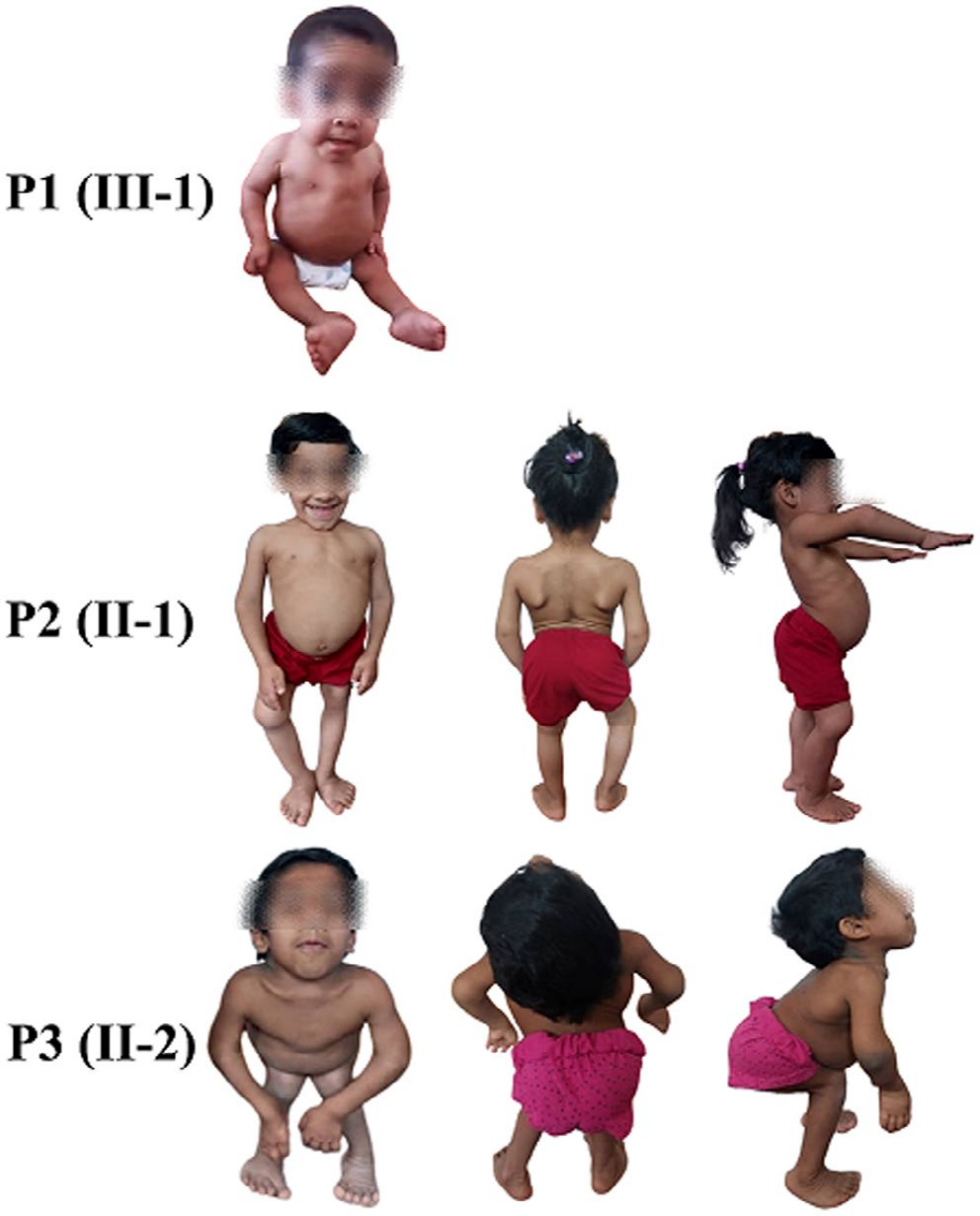
Clinical photographs of Patients P1 (III‐1), P2 (II‐1), and P3 (II‐2). P1 (III‐1): A 2.5‐year‐old boy. The patient exhibits short stature, pectus excavatum, cubitus valgus, and genu valgum. The hands and feet display brachydactyly with small nails. Facial features include a high anterior hairline, sparse scalp hair, broad and medially sparse eyebrows, and retrognathia. Additionally, the fingers and toes demonstrate joint hypermobility. P2 (II‐1): An 8‐year‐old girl is the maternal aunt of P (I‐1) and presents with more pronounced symptoms, such as congenital hip dislocation and short stature. P3 (II‐2). A 13.5‐year‐old girl is the maternal aunt of P (I‐1) and has notable characteristics, including short stature, disproportionately short limbs, and joint laxity. P2 (II‐1) and P3 (II‐2) have hand abnormalities, including brachydactyly (shortened fingers due to abnormal growth of phalanges) and underdeveloped or small nails. Proportional discrepancies in the lengths of fingers and phalanges and possible clinodactyly (curving of digits, typically the fifth finger) further characterize the hand dysmorphology associated with this rare skeletal dysplasia.

A consanguineous family from the Fars‐speaking community in Kerman province, located in central Iran, was referred for genetic evaluation due to the presence of multiple individuals with severe short stature and skeletal abnormalities across several generations. The pedigree suggested an autosomal recessive pattern of inheritance (Figure 1). The proband (P1, III‐1) was a male child born to first‐cousin parents. According to parental report, his postnatal growth velocity plateaued around one year of age. Clinical examination revealed microcephaly (head circumference −3 to −4 SD), low birth weight (1.5 kg), short stature, motor delay, hypotonia, pes cavus, bilateral clubfoot, sparse scalp hair, trident hand configuration, midface hypoplasia, kidney stones, and disproportionately short limbs, consistent with a diagnosis of skeletal dysplasia (Figure 2). Despite the presence of significant skeletal abnormalities, cognitive function and basic communication skills were preserved. The proband died at 2.5 years of age due to recurrent respiratory infections. Owing to his early death, peripheral blood samples were not available for genetic analysis, and molecular confirmation was therefore performed in the surviving affected family members.

Subsequent clinical evaluation of extended family members identified two additional affected individuals. Patient P2 (II‐1), an 8‐year‐old female and maternal aunt of the proband, presented with marked short stature, congenital hip dislocation, thoracolumbar kyphoscoliosis, genu varum, joint laxity, and brachydactyly (Figure 2). Patient P3 (II‐2), a 13‐year‐old male and maternal uncle of the proband, exhibited microcephaly, short stature, disproportionately short limbs, joint laxity, pes cavus, genu varum, and brachydactyly. Anthropometric measurements for all affected individuals are summarized in Table 1.

**TABLE 1:** Clinical and anthropometric features of patients in the present study compared with previously reported ANXD3 cases [3, 4].

Features	Patient 1 (III‐1)	Patient 2 (II‐1)	Patient 3 (II‐2)	Narayanan et al. 2019	Remmelzwaa et al. 2023
Gender/Age	M/2.5 year	F/8 year	M/13 year	F/6 year	F/7 year
Ethnicity	Iranian	Iranian	Iranian	Arabian	Eastern Africa
Short stature	+	+	+	+	+
Low birth weight	1.5 kg	NA	NA	1.5 kg at 30 week	NA
Macrocephaly	−	−	−	−	+
Microcephaly	+	+	+	+	−
Head circumference at born (cm)	29 (−3 to −4 SD)	32 (−2 to −3 SD)	31 (−2 to −3 SD)	50 (−3 to −4 SD)	48 (−2 SD)
Sparse scalp hair	+	−	−	+	−
High anterior hairline	−	−	−	+	−
Midface hypoplasia	−	−	−	−	−
Broad eyebrows	−	−	−	+	−
Frequent lung infections	+	+	−	−	+
Narrow thorax	+	−	+	−	−
Pectus excavatum	+	−	+	+	−
Poor feeding	NA	NA	NA	−	NA
Gastroesophageal reflux	NA	NA	NA	−	NA
Joint hypermobility	NA	+	NA	+	+
Joint dislocations	−	+	−	+/Bilateral elbow	+
Platyspondyly	Na	NA	NA	−	+
Ovoid vertebral bodies	NA	NA	NA	+	+
Thoracolumbar kyphoscoliosis	NA	+	+	+	+
Small and squaring of ilia	NA	NA	NA	−	−
Narrow acetabular angle	+	+	+	+	−
Bowing of femur	NA	NA	NA	−	+
Irregular cupped metaphyses of long bones	NA	NA	NA	+	−
Brachydactyly	+	+	+	+	+
Short metacarpals	+	+	+	+	+
Short broad middle phalanges	+	+	+	+	+
Trident hand	NA	+	+	+	+
Short toes	+	+	+		+
Skin laxity	NA	+	+	+	+
Cognitive delay	NA	+	+	−	+
Motor delay	+	+	+	−	+
Hypotonia	+	−	−	−	−
Club foot	+	NA	+	−	−
pes cavus	+	+	+	+	−
Short lower limb	+	NA	NA	−	+
Wrist flexion	+	NA	+	−	−
genu varum	NA	+	+	+	+
Hip dislocation	NA	+	NA	+	+
Knee dislocation	NA	+	NA	+	+

*Note:* Head circumference is reported with SD scores when available.

Abbreviations: +, feature present; −, feature absent; NA, not assessed.

Family history revealed several additional relatives with comparable skeletal phenotypes. According to family reports, a paternal cousin of the proband presented prenatally with severe cranial abnormalities and hydrops fetalis, resulting in intrauterine fetal demise. Furthermore, distant relatives (individuals II‐4 to II‐7) were reported to have exhibited short stature and skeletal deformities, with some affected individuals dying in early adulthood. Detailed clinical records for these individuals were not available.

## 3 Methods (Differential Diagnosis, Investigations and Treatment)

### 3.1 Investigations

This study was conducted in accordance with the tenets of the Declaration of Helsinki. Genomic DNA was extracted from the peripheral blood leukocytes of all available participants. As the proband (P1) died at 2.5 years of age, no peripheral blood sample was available for genetic analysis. Trio whole‐exome sequencing of the affected surviving individual(s) and the parents was conducted using the SureSelect Human All Exon V7 kit (Agilent) for exonic sequence capture and the Illumina NovaSeq 6000 platform for sequencing, achieving a mean coverage of > 150×. The sequences were aligned to the reference human genome (GRCh38/hg38).

We employed standard pipelines for analyzing Fastq, BAM, and BAI files. Variant calling was conducted using count and mean/median depth of variants classified by Callers, Zygosity GATK (3.2.2) and DeepVariant tools for more confidence. The vcf files were annotated using ANNOVAR and our custom annotation tool. Subsequently, variant filtering and variant curation were conducted using the selected databases as follows: The 1000 Genomes project (https://www.internationalgenome.org/), Exome Aggregation Consortium database (https://exac.broadinstitute.org/), the Genome Aggregation Database (gnomAD) (https://gnomad.broadinstitute.org/), NHLBI GO Exome Sequencing Project (ESP) (https://esp.gs.washington.edu/drupal/), Iranome (https://www.iranome.com/), and our in‐house database including 2500 individuals. We recruited ACMG guideline and Clinvar database (https://www.ncbi.nlm.nih.gov/clinvar) (access date: November 2023) for variant classification and used the ACMG SFv3.0 for considering secondary findings [6]. In addition, we conducted CNV calling after normalization of data using our in‐house database. The reads of SNPs and Indels are mapped through UCSC Genome Reference Consortium Human Build 38 (GRCh38), and variant calling is performed accordingly [7]. As an orthogonal validation for discovered variants, we recruited Sanger sequencing for the region of interest in the *NEPRO* gene.

To perform structural modeling, the target protein amino acid sequence was obtained from the UniProt database in FASTA format (https://www.uniprot.org/). Protein structure prediction was done using SWISS‐MODEL (https://swissmodel.expasy.org/), an automated homology‐based protein structure modeling server. The target protein sequence was uploaded to SWISS‐MODEL, which searched for homologous structures in the PDB and generated the predicted structure accordingly. Genomic data for the study were obtained from the DECIPHER database (https://www.deciphergenomics.org/), a comprehensive platform for analyzing and interpreting genomic data, particularly focused on rare genetic disorders.

### 3.2 Treatment

Postoperative recovery was supported by structured physiotherapy regimens tailored to enhance joint mobility, strengthen periarticular musculature, and improve overall functional outcomes. The physiotherapeutic approach was designed in alignment with standard protocols for post‐hip surgery rehabilitation in skeletal dysplasia patients.

## 4 Conclusion and Results (Outcome and Follow‐Up)

Pedigree analysis supported an autosomal recessive mode of inheritance. Whole‐exome sequencing identified a homozygous pathogenic variant in *NEPRO* (Chr3:113014014G>A; NM_015412.4:c.280C>T; p.Arg94Cys) in the proband. Sanger sequencing confirmed heterozygous carrier status in both parents and co‐segregation of the variant with the disease phenotype within the family (Figure 1). These findings established the molecular diagnosis of anauxetic dysplasia type 3 (ANXD3) in the affected individuals.

Radiographic images were not available for review, and this limitation is acknowledged.

**FIGURE 3:**
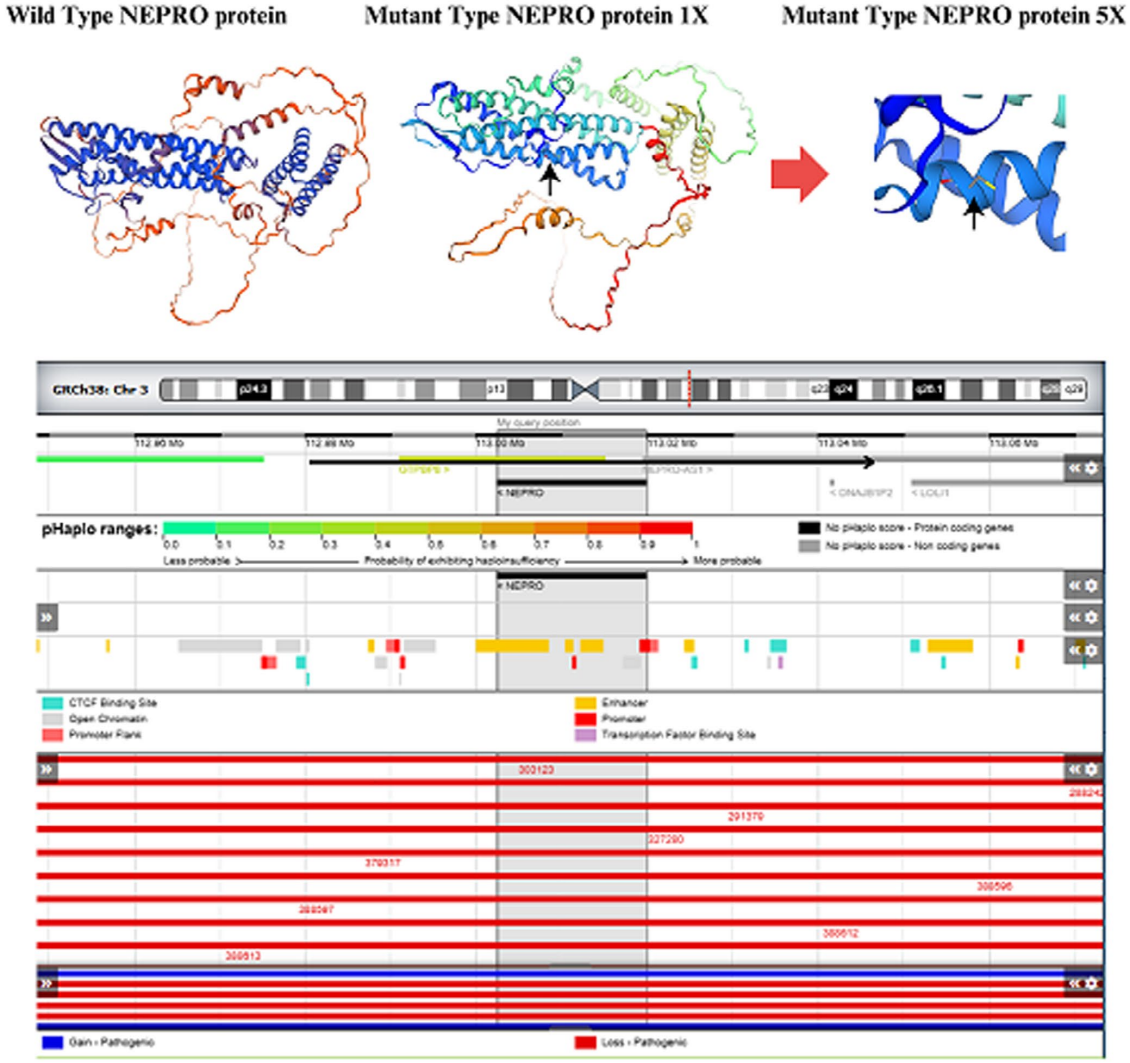
Genomic annotation of the NEFRO gene region on Chromosome 3 (GRCh38). (A) Genomic landscape of the NEPRO locus on chromosome 3 (GRCh38). The gene is located within a region enriched in regulatory elements, including CTCF binding sites (purple), open chromatin regions (blue), and promoter flanking regions (green), indicating regulatory complexity. Pathogenic gain variants are shown in red and pathogenic loss variants are shown in orange. No haploinsufficiency score has been reported for NEPRO, suggesting no established association with dosage‐sensitive disorders. (B) Predicted three‐dimensional structure of the NEPRO protein generated using AlphaFold‐based modeling. The structure is colored according to model confidence (blue: High confidence; yellow/orange: Lower confidence regions). (C) Magnified view of residue Arg94 within the predicted structure. The R94C substitution identified in this family (c.280C>T; p.Arg94Cys) is shown in stick representation and indicated by a black arrow to clearly highlight its spatial position within the protein structure. The structural model is included to provide spatial context for the recurrent R94C variant previously reported in ANXD3, rather than as functional validation. Together, these panels illustrate both the regulatory genomic environment and the structural localization of the identified pathogenic variant.

The *NEPRO* gene, located on chromosome 3 (GRCh38), is a protein‐coding gene situated within a genomic region enriched in regulatory elements. Although no haploinsufficiency score has been formally reported—indicating no established association with dosage‐sensitive disorders—the locus is surrounded by multiple regulatory features, including CTCF binding sites, open chromatin regions, and promoter flanking elements. This regulatory landscape suggests potential complexity in transcriptional control. In addition, both pathogenic gain‐of‐function and loss‐of‐function variants have been mapped in proximity to the NEPRO locus, which may have implications for disease mechanisms or phenotypic variability (Figure 3).

This study expands the phenotypic spectrum of ANXD3 by reporting three new cases with unique systemic features, including microcephaly, clubfoot, cataracts, urolithiasis, and hearing impairments. The identification of a homozygous *NEPRO* variant in these patients reinforces the genetic basis of ANXD3 and highlights the importance of considering systemic involvement in the clinical management of this disorder. The findings underscore the need for a multidisciplinary approach to care, involving orthopedic, ophthalmologic, audiologic, and renal specialists, to address the diverse manifestations of ANXD3. Continued research into the molecular mechanisms underlying this disorder will be essential for developing targeted therapies and improving outcomes for affected individuals.

## 5 Discussion

This study presents three new cases of anauxetic dysplasia type 3 (ANXD3) from a consanguineous Iranian family, expanding the phenotypic spectrum of this rare autosomal recessive skeletal dysplasia. All patients carried a homozygous pathogenic variant in the NEPRO gene (Chr3:113014014G>A; c.280C>T; p.Arg94Cys), which is consistent with the severe skeletal manifestations typically observed in ANXD3, including short stature, brachydactyly, and joint hypermobility.

Comparison with previously reported cases by Narayanan et al. (2019) and Remmelzwaal et al. (2023) demonstrates both shared and distinct features. All patients consistently exhibited severe short stature and brachydactyly, whereas systemic manifestations and craniofacial features varied, reflecting the phenotypic heterogeneity of ANXD3. The occurrence of urolithiasis in one patient, although not previously reported, is noted as an observation requiring further evaluation in larger cohorts [3, 4].

Overall, these findings underscore the importance of careful phenotypic characterization in ANXD3 and support the value of comprehensive genetic analysis for diagnosis and family counseling. Moreover, the variability observed emphasizes the need for individualized clinical management, particularly for orthopedic follow‐up, while potential extra‐skeletal features should be considered cautiously until confirmed by further studies.

## 6 Clinical Presentation and Genetic Insights

The clinical presentation of the three patients in this study is consistent with the severe skeletal dysplasia characteristic of ANXD3, as previously described in the literature. Radiographic findings, including ovoid vertebrae, hypoplastic ilia, and narrow acetabular angles, further support the diagnosis. In addition to the core skeletal manifestations, several additional clinical features were observed in this family, including microcephaly and clubfoot, which may contribute to a broader phenotypic spectrum of ANXD3.

Review of available medical records for one affected individual who died in early childhood revealed a suspected cataract and a possible unilateral hearing impairment; however, these findings were not confirmed by formal ophthalmologic or audiologic evaluations. Therefore, these features were not considered part of the definitive core phenotype in the affected living patients but are noted as observations that may warrant further investigation in future cases. Collectively, these findings suggest that NEPRO gene variants may have pleiotropic effects, potentially impacting systems beyond skeletal development [1, 3, 4, 8].

The identification of a homozygous NEPRO gene variant in this family provides further evidence of the involvement of this gene in skeletal development. NEPRO encodes a protein associated with ribosomal RNA processing, and the identified variant (p.Arg94Cys) is predicted to interfere with this process, which may contribute to the observed skeletal abnormalities. This observation is consistent with previously reported NEPRO variants in patients with ANXD3 and reinforces the genetic basis of this disorder [3].

One notable aspect of this study is the phenotypic variability observed among affected individuals within the same extended family. Although all patients exhibited the hallmark skeletal features of ANXD3, variability was observed in the presence and severity of associated findings, including joint laxity, spinal deformities, and extra‐skeletal manifestations. Recurrent urolithiasis observed in one patient may suggest possible renal involvement; however, given the limited number of affected individuals, this finding should be interpreted with caution.

Comparative analysis with previously reported ANXD3 cases described by Narayanan et al. (2019) and Remmelzwaal et al. (2023) [3, 4] demonstrates both overlapping and distinct clinical features. Severe short stature, brachydactyly, and thoracolumbar kyphoscoliosis were consistently observed across all reported patients, supporting their role as core features of ANXD3. In contrast, systemic involvement and craniofacial features showed considerable variability.

With respect to head circumference, the Iranian patients presented with microcephaly, whereas macrocephaly was reported in patients described by Narayanan et al. and Remmelzwaal et al. [3, 4]. This discrepancy suggests that NEPRO variants may exert variable effects on cranial development, potentially influenced by genetic background, modifier genes, or environmental factors. Sparse scalp hair was observed in one Iranian patient and in Narayanan's case but was absent in the patient reported by Remmelzwaal et al., further illustrating phenotypic heterogeneity.

Neurodevelopmental outcomes also varied among reported cases. Cognitive and motor delays were observed in the Iranian patients and in the case reported by Remmelzwaal et al., whereas Narayanan's patient did not exhibit developmental delay. These differences may reflect variability in disease severity or the influence of additional modifying factors. Certain skeletal features, such as irregular cupped metaphyses and femoral bowing reported by Remmelzwaal et al., and bilateral elbow joint involvement reported by Narayanan et al., were not observed in the present family (Table 1), underscoring the broad clinical spectrum of ANXD3 [3, 4].

The molecular diagnosis of ANXD3 in this family was established using whole‐exome sequencing followed by segregation analysis with Sanger sequencing. This approach highlights the importance of comprehensive genetic testing in the evaluation of rare skeletal dysplasia, particularly in consanguineous families where autosomal recessive inheritance is more prevalent. Identification of the NEPRO variant enabled confirmation of the diagnosis and provided essential information for genetic counseling.

From a clinical perspective, the findings in this family emphasize the importance of multidisciplinary follow‐up. Orthopedic manifestations such as clubfoot and hip dislocation require early intervention to optimize mobility. Although ocular and auditory abnormalities were not definitively confirmed in the living patients, these observations suggest that targeted ophthalmologic and audiologic assessments may be considered during follow‐up. Additionally, the occurrence of urolithiasis highlights the potential value of renal monitoring and metabolic evaluation in selected cases (Figure 1).

## 7 Limitations and Future Directions

This study has several limitations. First, the unavailability of blood samples from deceased family members limited the genetic analysis to living patients. Additionally, the lack of radiographic images due to the family's refusal to undergo anesthesia for imaging procedures restricted the ability to fully characterize the skeletal abnormalities in these patients. Future studies should aim to include radiographic and histopathological analyses to provide a more comprehensive understanding of the skeletal and systemic manifestations of ANXD3.

Further research is also needed to elucidate the molecular mechanisms underlying the systemic features observed in ANXD3 patients. Understanding how *NEPRO* variants impact ribosome biogenesis and lead to these diverse clinical manifestations could provide insights into potential therapeutic targets. Additionally, longitudinal studies are needed to assess the long‐term outcomes of ANXD3 patients and to develop evidence‐based guidelines for their management.

## Author Contributions

**Mahnaz Mohammadi Kian:** methodology, writing – original draft. **Sara Sheikholeslami:** visualization, writing – review and editing. **Maryam Kiani Feizabadi:** investigation. **Amir Mohammad Roshanaeizadeh:** software, validation. **Atousa Haghi:** software. **Ahmad Reza Salehi Chaleshtori:** data curation, supervision. **Hamid Reza Moazzeni:** supervision.

## Funding

The authors have nothing to report.

## Consent

Written informed consent was obtained from the patient's parents to publish this report following the journal's consent policy.

## Conflicts of Interest

The authors declare no conflicts of interest.

## Data Availability

The data that support the findings of this study are available on request from the corresponding author. The data are not publicly available due to privacy or ethical restrictions.
